# Balanced Crystalloids for Pediatric Sepsis and Septic Shock

**DOI:** 10.1111/acem.70115

**Published:** 2025-07-28

**Authors:** Brit Long, Michael Gottlieb

**Affiliations:** ^1^ Department of Emergency Medicine University of Virginia Medical Center Charlottesville Virginia USA; ^2^ Department of Emergency Medicine Rush University Medical Center Chicago Illinois USA

**Keywords:** acute kidney injury, balanced crystalloids, balanced fluids, infectious disease, normal saline, pediatric sepsis, pediatric septic shock, pediatrics, renal replacement therapy

**Table:** 

NNT color recommendation	Yellow (unclear if benefits)
Summary heading	Balanced crystalloids reduced the risk of renal replacement therapy requirement when compared to normal saline
Benefits in NNT	Balanced crystalloids compared to normal saline:
	– 1 in 18 were helped (the need for renal replacement therapy prevented)
	– No one was helped (no acute kidney injury prevented)
Benefits in percentages (absolute risk reduction)	– 5.9% reduction in risk of renal replacement therapy requirement
	– No reduction in the risk of acute kidney injury
Harms in NNT (NNH)	Not reported
Harms in percentages	Not reported
Efficacy endpoints	Acute kidney injury, hospital mortality, need for renal replacement therapy, hyperchloremia, length of hospital and pediatric intensive care unit stay, and mechanical ventilation
Harm endpoints	Not reported
Who was in the studies	Eight total studies with four randomized trials comprising 861 participants < 18 years with septic shock

## 1 Narrative

Septic shock is a complication of sepsis associated with circulatory dysfunction and multiorgan injury [1, 2, 3]. Morbidity and mortality from sepsis are high. Sepsis accounts for 19% of global deaths, and the highest incidence is in children under 5 years old [4]. Source control, antibiotics, and resuscitation with intravenous (IV) fluids are essential components of management [1, 3]. Options for IV fluids include balanced crystalloids (e.g., PlasmaLyte, Lactated Ringers [LR]) and normal saline. Currently, there is controversy regarding which class of fluids to utilize for resuscitation [1, 3, 5, 6]. Studies suggest that normal saline may contribute to hyperchloremia, acute kidney injury (AKI), and increased inflammation, as normal saline has higher levels of sodium and chloride compared to balanced crystalloids which more closely resemble the composition of serum [5, 6, 7, 8, 9, 10]. However, the evidence for the use of balanced crystalloids has not been strong [5, 6, 7, 8, 9, 10, 11]. Here we summarize a systematic review and meta‐analysis that assessed the effectiveness of balanced crystalloids versus normal saline for resuscitation of pediatric patients (< 18 years) with sepsis or septic shock [12].

The systematic review and meta‐analysis discussed here included 8 studies of pediatric patients with sepsis or septic shock (*n* = 12,231 participants), with 4 randomized controlled trials (RCTs) (*n* = 861 participants) [12]. The systematic review included studies evaluating children 0–18 years diagnosed with sepsis or septic shock requiring fluid resuscitation which compared balanced crystalloids versus normal saline. Authors included randomized and nonrandomized trials reporting at least one outcome of interest. The mean age of participants ranged from 2.4 to 10.4 years, and females accounted for 42%–53% of included patients. The mean Pediatric Risk of Mortality III (PRISM III) score ranged from 5 to 8.75. A respiratory infection was the underlying etiology of sepsis in 16.1%–58.7% of patients. Two studies used a combination of LR and PlasmaLyte or Sterofundin, three studies used only LR, two studies used PlasmaLyte or Ringer's acetate, and one study did not report the type of balanced crystalloid.

The outcomes of interest were AKI defined based on the Kidney Disease: Improving Global Outcomes (KDIGO) or International Classification of Diseases, Ninth Revision (ICD‐9) codes; hospital mortality; hospital length of stay (LOS); pediatric intensive care unit LOS; need for renal replacement therapy (RRT); mechanical ventilation; and hyperchloremia. The KDIGO definition of AKI includes three stages [13]. Stage 1 is an increase in serum creatinine of > 0.3 mg/dL within 48 h or an increase in creatinine of ≥ 50% within 7 days, with urine output > 0.5 and < 1 mL/kg/h. Stage 2 is an increase in creatinine of ≥ 100%, with urine output > 0.3 and < 0.5 mL/kg/h. Stage 3 is an increase in creatinine of ≥ 200%, serum creatinine > 4 mg/dL, receipt of dialysis, or estimated glomerular filtration rate < 35 mL/min/1.73 m^2^, with urine < 0.3 mL/kg/h [13]. RRT was defined as hemodialysis, continuous renal replacement, hemofiltration and hemodiafiltration, peritoneal dialysis, and kidney transplantation.

For the purpose of this review, we focus on the subgroup analysis that only included pooled data from RCTs. Compared to normal saline, balanced crystalloids were associated with a lower risk of RRT requirement (risk ratio [RR]: 0.58; 95% confidence interval [CI], 0.39 to 0.87; absolute risk difference [ARD]: 5.9%; number needed to treat [NNT]: 18) [12].

Combining observational and randomized data, the systematic review reported a lower risk of hyperchloremia (RR: 0.70; 95% CI, 0.59 to 0.82) but a longer hospital LOS (mean difference: 3.38 days; 95% CI, 1.13–5.64 days) with balanced crystalloids compared to normal saline. There was no difference in AKI, mortality, mechanical ventilation, or pediatric intensive care unit LOS [12].

## 2 Caveats

There are several important limitations to this systematic review [12]. First, the meta‐analysis included observational studies and RCTs, and two retrospective studies accounted for the majority of the patients included in the systematic review [14, 15]. This led to a significant risk of confounding. We opted to focus on RCT data to mitigate the risk of bias. However, the RCT data were limited by small sample sizes, which may not have had sufficient power to detect clinically significant differences. Second, the included studies evaluated a wide range of ages (birth to 18 years), though only two studies stratified outcomes based on age groups. Authors also included studies evaluating both sepsis and septic shock, which vary in the degree of illness severity. Focusing on studies with a greater composition of septic shock patients or including only studies with septic shock may yield a more significant result in favor of balanced crystalloids. Third, there was significant variability in the included studies concerning the etiology of sepsis, the outcomes evaluated, the definition of AKI, and follow‐up periods, contributing to heterogeneity. There was also limited reporting of outcomes based on overall fluid balance.

Based on the available data from this systematic review, the use of balanced crystalloids in pediatric sepsis and septic shock likely reduces the need for RRT. However, due to the high risk of bias resulting from including observational and retrospective studies, the validity of these results are questionable. There is likely no difference in mortality, and the small sample sizes do not provide sufficient power to detect clinically significant differences.

Of note, a 2025 meta‐analysis including 5 RCTs (*n* = 992) evaluated balanced crystalloids and normal saline in children 1 month to 18 years [16]. They found balanced crystalloids were associated with a lower risk of AKI (RR: 0.64; 95% CI, 0.50 to 0.8), RRT (RR: 0.52; 95% CI, 0.35 to 0.76), and hyperchloremia (RR: 0.74, 95% CI, 0.62 to 0.87). However, they did not find a difference in mortality, and the overall risk of bias was low and unclear in most domains, which led to a low certainty level rating for these findings [16]. As of the publication of this manuscript, both the adult and pediatric Surviving Sepsis Campaign International Guidelines recommend the use of balanced crystalloids over normal saline [1, 17].

In summary, while balanced crystalloids may offer some benefits over normal saline in pediatric patients with sepsis and septic shock, the evidence comes from a small sample of RCTs as well as observational studies and requires confirmation through larger studies. Future research should also assess other patient‐centered outcomes such as mortality to ensure the robustness of these benefits. As such, we have assigned a Yellow (unclear benefit; more data needed) NNT color recommendation to this intervention.

## Conflicts of Interest

The authors declare no conflicts of interest.

## Data Availability

Data sharing not applicable to this article as no datasets were generated or analysed during the current study.
